# Pulmonary embolism in patients with COVID-19: characteristics and outcomes in the Cardio-COVID Italy multicenter study

**DOI:** 10.1007/s00392-020-01766-y

**Published:** 2020-11-03

**Authors:** Pietro Ameri, Riccardo M. Inciardi, Mattia Di Pasquale, Piergiuseppe Agostoni, Antonio Bellasi, Rita Camporotondo, Claudia Canale, Valentina Carubelli, Stefano Carugo, Francesco Catagnano, Giambattista Danzi, Laura Dalla Vecchia, Stefano Giovinazzo, Massimiliano Gnecchi, Marco Guazzi, Anita Iorio, Maria Teresa La Rovere, Sergio Leonardi, Gloria Maccagni, Massimo Mapelli, Davide Margonato, Marco Merlo, Luca Monzo, Andrea Mortara, Vincenzo Nuzzi, Massimo Piepoli, Italo Porto, Andrea Pozzi, Giovanni Provenzale, Filippo Sarullo, Gianfranco Sinagra, Chiara Tedino, Daniela Tomasoni, Maurizio Volterrani, Gregorio Zaccone, Carlo Mario Lombardi, Michele Senni, Marco Metra

**Affiliations:** 1 IRCCS Ospedale Policlinico San Martino and Department of Internal Medicine, University of Genova, Genova, Italy; 2 Cardiology, ASST Spedali Civili and Department of Medical and Surgical Specialties, Radiological Sciences and Public Health, University of Brescia, Brescia, Italy; 3 Centro Cardiologico Monzino, IRCCS, Milan, Italy; 4 Innovation and Brand Reputation Unit, Papa Giovanni XXIII Hospital, Bergamo, Italy; 5 Fondazione IRCCS Policlinico S. Matteo and University of Pavia, Pavia, Italy; 6 Division of Cardiology, Ospedale San Paolo, ASST Santi Paolo E Carlo, University of Milano, Milan, Italy; 7 Cardiology Department, Policlinico Di Monza, Monza, Italy; 8 Dipartimento Di Cardiologia, Istituti Clinici Scientifici Maugeri, IRCCS, Istituto Scientifico Di Milano, Milan, Italy; 9 Division of Cardiology, Ospedale Maggiore Di Cremona, Cremona, Italy; 10 Heart Failure Unit, Cardiology Department, IRCCS San Donato Hospital, University of Milan, Milan, Italy; 11 Cardiovascular Department and Cardiology Unit, Papa Giovanni XXIII Hospital-Bergamo, Piazza OMS, 1, 24127 Bergamo, Italy; 12 Dipartimento Di Cardiologia, Istituti Clinici Scientifici Maugeri, IRCCS, Istituto Scientifico Di Pavia, Pavia, Italy; 13 Cardiovascular Department, Azienda Sanitaria Universitaria Integrata, Trieste, Italy; 14 Istituto Clinico Casal Palocco, Rome, Italy; 15 Heart Failure Unit, G da Saliceto Hospital, AUSL Piacenza, Piacenza, Italy; 16 Institute of Life Sciences, Sant’Anna School of Advanced Studies, Pisa, Italy; 17 Cardiovascular Rehabilitation Unit, Buccheri La Ferla Fatebenefratelli Hospital, Palermo, Italy; 18 Department of Medical Sciences, Istituto Di Ricovero E Cura a Carattere Scientifico (IRCCS) San Raffaele Pisana, Rome, Italy; 19 Policlinico Casilino, Rome, Italy; 20 Department of Clinical Sciences and Community Health, University of Milano, Milan, Italy

**Keywords:** COVID-19, Thromboembolism, d-dimer, Coagulopathy, Anticoagulant, Death

## Abstract

**Background:**

Pulmonary embolism (PE) has been described in coronavirus disease 2019 (COVID-19) critically ill patients, but the evidence from more heterogeneous cohorts is limited.

**Methods:**

Data were retrospectively obtained from consecutive COVID-19 patients admitted to 13 Cardiology Units in Italy, from March 1st to April 9th, 2020, and followed until in-hospital death, discharge, or April 23rd, 2020. The association of baseline variables with computed tomography-confirmed PE was investigated by Cox hazards regression analysis. The relationship between d-dimer levels and PE incidence was evaluated using restricted cubic splines models.

**Results:**

The study included 689 patients (67.3 ± 13.2 year-old, 69.4% males), of whom 43.6% were non-invasively ventilated and 15.8% invasively. 52 (7.5%) had PE over 15 (9–24) days of follow-up. Compared with those without PE, these subjects had younger age, higher BMI, less often heart failure and chronic kidney disease, more severe cardio-pulmonary involvement, and higher admission d-dimer [4344 (1099–15,118) vs. 818.5 (417–1460) ng/mL, *p* < 0.001]. They also received more frequently darunavir/ritonavir, tocilizumab and ventilation support. Furthermore, they faced more bleeding episodes requiring transfusion (15.6% vs. 5.1%, *p* < 0.001) and non-significantly higher in-hospital mortality (34.6% vs. 22.9%, *p* = 0.06). In multivariate regression, only d-dimer was associated with PE (HR 1.72, 95% CI 1.13–2.62; *p* = 0.01). The relation between d-dimer concentrations and PE incidence was linear, without inflection point. Only two subjects had a baseline d-dimer < 500 ng/mL.

**Conclusions:**

PE occurs in a sizable proportion of hospitalized COVID-19 patients. The implications of bleeding events and the role of d-dimer in this population need to be clarified.

**Graphic abstract:**

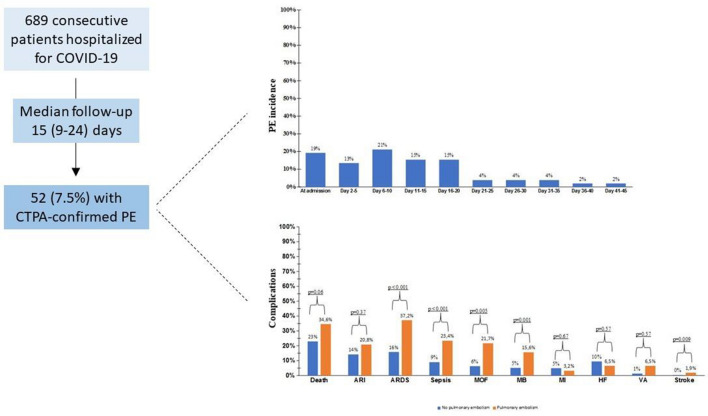

**Electronic supplementary material:**

The online version of this article (doi:10.1007/s00392-020-01766-y) contains supplementary material, which is available to authorized users.

## Introduction

Pneumonia is the major clinical manifestation of novel coronavirus disease 2019 (COVID-19) caused by severe acute respiratory syndrome coronavirus 2 (SARS-CoV-2) [1, 2]. Nonetheless, the cardiovascular (CV) system is often affected in COVID-19 [3]. Abnormalities in laboratory parameters indicating activation of the coagulation cascade are frequent in patients with COVID-19, and have been related to SARS-CoV-2-initiated inflammatory response and endothelial injury [4]. At present, the main consequence of COVID-19 coagulopathy appears to be arterial and especially venous thromboembolism (VTE), with the lung being the organ most commonly involved. Autopsy studies have demonstrated the presence of thrombi in the pulmonary arteries and alveolar capillaries of individuals deceased from COVID-19 [5, 6]. Clinically, 20–30% of COVID-19 patients admitted to intensive care units (ICU) have pulmonary embolism (PE) [7–10]. The frequency of PE in subjects with non-critical COVID-19 seems to be lower, but it has far less been studied [11–13]. Importantly, the risk of VTE is influenced by patient- and care-related factors, such as ethnicity and intensity of treatments [4]. Moreover, the definite diagnosis of PE relies on computed tomography pulmonary angiography (CTPA), which has likely been accessible to a variable extent in different hospitals even within the same country, due to local measures adopted to limit the spreading of SARS-CoV-2. Hence, it is important to obtain data about PE in COVID-19 from heterogeneous cohorts of patients, in order to expand the knowledge and possibly inform clinical practice.

To this scope, we reviewed the dataset created by a collaborative initiative of several Cardiology Units involved in the management of COVID-19 patients in Italy.

## Methods

### Study population

This retrospective, multicenter, observational study included consecutive patients with laboratory-confirmed COVID-19 admitted to 13 Cardiology Units in Italy from March 1st to April 9th, 2020, regardless of concomitant cardiac disease. Supplemental Fig. 1 displays the location of the centers across Italy: those in the Lombardy region accounted for 524 (76.1%) patients. In total, 7670 subjects with SARS-CoV-2 infection were admitted to the hospitals of the participating Cardiology Units during the study period.

Diagnosis of COVID-19 was based on suggestive clinical presentation, evidence of pneumonia at the chest radiography or CT, and amplification of SARS-CoV-2 RNA by real time reverse transcriptase–polymerase chain reaction of pharyngeal swabs or lower respiratory tract aspirates.

The study complied with the Declaration of Helsinki and was approved by the ethical committee of Spedali Civili di Brescia, Brescia, Italy (no. NP 4105). Informed consent was obtained from all patients who could give it. Only de-identified (anonymized) data were analyzed.

### Data collection

Medical records of the subjects included in the study were reviewed until in-hospital death, discharge, or April 23rd, 2020, so that the minimum duration follow-up of alive patients was 14 days. Those who were no longer hospitalized at the end of the follow-up are hereafter defined as closed cases. The following information was collected: PE, as diagnosed by CTPA; demographics and medical history; prior chronic medical therapy; clinical features, laboratory exams and right ventricle (RV)-focused echocardiography measures at the time of admission to hospital; non-CV and CV complications that occurred during the hospitalization; type of ventilation support and drugs received for COVID-19; and in-hospital death. Preexisting diseases and complications of COVID-19 were assigned if reported in the medical records, with no specific definition. In the analysis, bleeding events requiring transfusion were considered as major. Concentrations of d-dimer 2 days after admission, at peak and at discharge were also retrieved. Beyond the d-dimer cut-off of 500 ng/mL, the one calculated as age × 10 ng/mL was taken into account for > 50 year-old subjects [14].

In the effort to better characterize the context in which PE occurred, the medical records of patients with PE were further reviewed and additional information was obtained about anticoagulant therapy and risk factors for VTE.

### Statistical analysis

Continuous variables are presented as mean with standard deviation (SD) if normally distributed and median with interquartile range if skewed, dichotomous variables as count and percentage of total. Comparisons between groups were made by Student’s *t* test for normally distributed continuous variables, Wilcoxon test for non-normally distributed ones, and Chi-squared test for proportions.

The frequency and incidence rate (with 95% confidence intervals, CI) of PE were calculated both in the whole cohort and among closed cases.

Proportional hazards regression was used to investigate the baseline variables associated with PE. First, univariate hazard ratios (HR) with 95% CI were computed for the variables significantly different between subjects without and with PE. Next, a stepwise forward selection was done to identify the correlates of PE. A sensitivity analysis using a backward selection was also performed. Furthermore, the multivariable model was also tested after substituting peak d-dimer concentrations for baseline ones.

The flexible continuous relationship between baseline and peak d-dimer levels and the incidence of PE was displayed using restricted cubic splines models with three knots, resulting in the lowest model Akaike information criterion (3–6 knots were assessed). The changes in d-dimer values throughout hospitalization in patients without vs. with PE were assessed using a mixed-effects longitudinal model.

Analyses were performed with Stata, version 14 (Stata Corp., College Station, TX, USA) and a *p* value < 0.05 was considered significant.

## Results

The study included 689 COVID-19 patients, of whom 52 (7.5%) were diagnosed with PE by CTPA over 15 (9–24) days of follow-up. The corresponding incidence rate was 4.6 (95% CI 3.5–6.1) per 1000 person-months. The median time from admission to diagnosis was 10 (3–17) days. Ten (19.2%) PE were found on the day of hospital entry and the other ones throughout hospitalization (Supplemental Fig. 2). 91 (13.2%) subjects were still hospitalized at the end of follow-up, with 599 (86.9%) being therefore closed cases. The frequency and incidence rate of PE among these latter were 7.1% and 5.2 (95% CI 3.9–7.0) per 1000 person-months, respectively.

### Patients’ characteristics and clinical course stratified by pulmonary embolism

The baseline clinical characteristics and main laboratory data of the study population are presented in Tables 1 and 2, respectively, according to the absence or occurrence of PE. Additional laboratory values are provided in Supplemental Table 1.

**Table 1:** Baseline characteristics of the study population, stratified by occurrence of pulmonary embolism

	All (*n* = 689)	No PE (*n* = 637)	PE (*n* = 52)	*p* value
Age (years)	67.3 ± 13.2	67.6 ± 13.4	63.8 ± 10.6	0.04
Male gender	487 (69.4)	437 (68.6)	41 (78.8)	0.12
BMI (kg/m^2^)	27.2 ± 5.3	27 ± 5.2	29.6 ± 6.3	0.003
Ever smoker	159 (27)	151 (27.7)	8 (18.6)	0.20
Hypertension	398 (56.9)	364 (57.6)	25 (48.1)	0.18
Dyslipidaemia	188 (27.5)	175 (27.7)	13 (25.0)	0.74
Diabetes	157 (23)	144 (22.8)	13 (25.0)	0.72
Heart failure	92 (13.5)	90 (14.2)	2 (3.8)	0.04
Atrial fibrillation	105 (15.4)	102 (16.1)	3 (5.8)	0.05
Coronary artery disease	143 (20.9)	137 (21.7)	6 (11.5)	0.08
COPD	67 (9.8)	64 (10.1)	3 (5.8)	0.31
Chronic kidney disease	127 (18.6)	123 (19.5)	4 (7.7)	0.04
ACEi/ARB therapy	133 (20.6)	123 (20.6)	10 (20.0)	0.91
Oral anticoagulant therapy	90 (14.1)	79 (13.5)	11 (21.6)	0.11
Direct oral anticoagulant	47 (7.4)	40 (6.8)	7 (13.7)	0.07
Vitamin K antagonist	48 (7.5)	43 (7.3)	5 (9.8)	0.52
Statin therapy	176 (27.2)	165 (27.7)	11 (21.6)	0.35
Fever (≥ 37.5 °C)	440 (64.1)	408 (64.3)	32 (62.7)	0.83
Respiratory rate ≥ 22/min	279 (52.0)	253 (50.8)	26 (66.7)	0.06
SBP (mmHg)	129.6 ± 21.5	129.7 ± 21.4	129.2 ± 22.4	0.89
Heart rate (bpm)	86.6 ± 18.1	86.3 ± 18.2	90.7 ± 15.9	0.09
Oxygen saturation (%)	90.5 ± 7.6	90.8 ± 7.2	86.6 ± 10.1	< 0.001
LV ejection fraction (%)	52.5 ± 11.3	52.1 ± 11.7	55.3 ± 8.4	0.12

Data are shown as count (%), mean ± SD or median (interquartile range)

*BMI* body mass index, *COPD* chronic obstructive pulmonary disease, *ACEi* angiotensin-converting enzyme inhibitor, *ARB* angiotensin receptor blocker, *SBP* systolic blood pressure, *LV* left ventricular

**Table 2:** Main laboratory findings on admission in the study population, stratified by occurrence of pulmonary embolism

	All (*n* = 689)	No PE (*n* = 637)	PE (*n* = 52)	*p* value
Hemoglobin (g/dL)	13.4 (11.8–14.4)	13.4 (11.8–14.4)	13.3 (12.1–14.9)	0.46
White blood cell count (/μL)	6760 (4990–9320)	6620 (4930–9200)	8500 (6717–11,525)	0.002
Lymphocyte count (/μL)	921.5 (620–1300)	921.5 (620–1,300)	932 (569–1200)	0.91
Platelet count (× 10^3^/μL)	204 (155–270)	201 (154–266)	242.5 (179.5–321)	0.01
Serum creatinine (mg/dL)	1.0 (0.8–1.3)	1.0 (0.8–1.3)	0.9 (0.9–1.4)	0.50
CRP (mg/dL)	233.5 (108.5–353.5)	238.5 (106.5–360.5)	203 (127.5–288.0)	0.22
Ferritin (μg/L)	698.5 (374–1473)	669 (368–1424)	1470 (729–1958)	0.01
Aspartate transaminase (U/L)	40 (26–64)	39 (25–63)	51.5 (36–82)	0.003
Lactate dehydrogenase (U/L)	362 (252–520)	353 (249–505)	459 (294–612)	0.03
d-dimer (ng/mL)	1,917 (820–5250)	818.5 (417–1460)	4,344 (1099–15,118)	< 0.001
INR	1.1 (1.0–1.2)	1.1 (1.0–1.2)	1.1 (1.0–1.3)	0.44
Elevated troponin	278 (45.3)	244 (44)	28 (59.6)	0.04
NT-proBNP (pg/mL)	341 (96–1323)	341 (96–1323)	333 (117–1183)	0.84

Data shown as median (interquartile range) or, for the frequency of elevated troponin, count (%)

*CRP* C-reactive protein, *INR* international normalized ratio, *NT-proBNP* N-terminal pro-B-type natriuretic peptide

Patients who developed PE were younger and had higher body mass index (BMI) than those without PE (Table 1). While CV risk factors were similarly distributed in the PE and no-PE groups, heart failure and chronic kidney disease were less common in the PE group. Anticoagulant therapy before hospitalization was numerically more frequent in patients with PE, primarily because of a larger use of direct oral anticoagulants (Table 1).

**Fig. 1:**
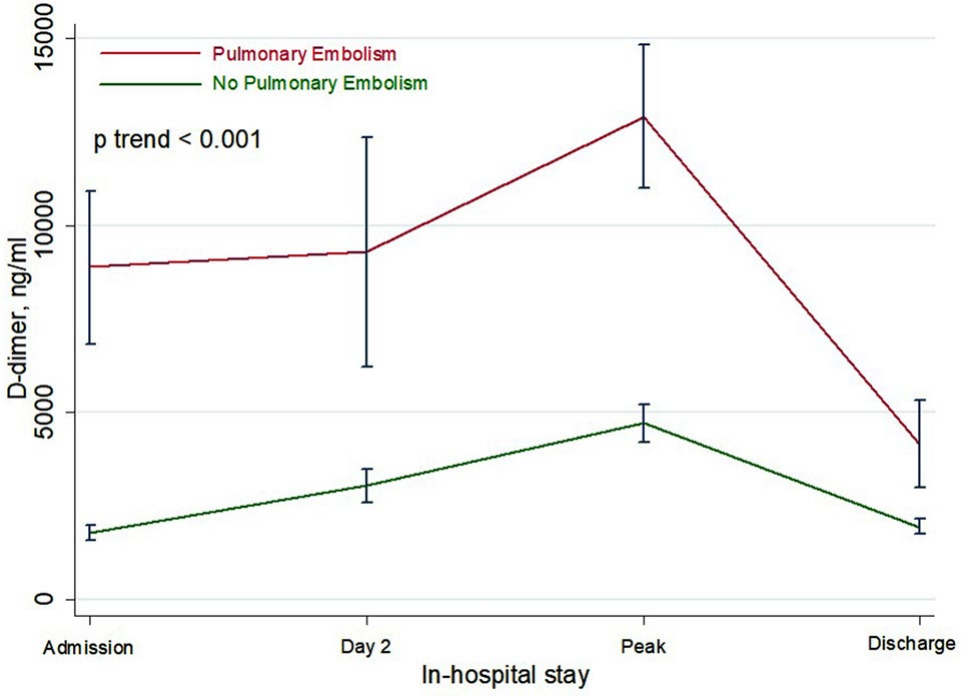
Concentrations of d-dimer at different time points throughout the hospitalization for COVID-19 in patients without or with pulmonary embolism

On admission, subjects with PE had a lower oxygen saturation than those without PE (Table1). As showed in Table 2, they also presented with higher white blood cell count and concentrations of ferritin, aspartate transaminase, lactate dehydrogenase and N-terminal pro-B-type natriuretic peptide (NT-proBNP); troponin levels were also more often above the upper normal limit in the PE group as compared with the one without PE. Among coagulation parameters, median d-dimer level on admission was fivefold higher in patients who suffered from PE than in those who did not; platelet count was also higher, while INR values were comparable (Table 2). Day 2 and peak concentrations of d-dimer were also higher in subjects with than without PE (3243 ng/mL vs. 1030 ng/mL and 5849 ng/mL vs. 1690 ng/mL, respectively; *p* < 0.001 for both comparisons). The temporal trend of d-dimer levels is displayed in Fig. 1.

The dataset contained RV echocardiographic parameters for 10–37% of the patients. With this limitation, RV dilation and dysfunction were more common in subjects with than without PE (Supplemental Table 2).

**Fig. 2:**
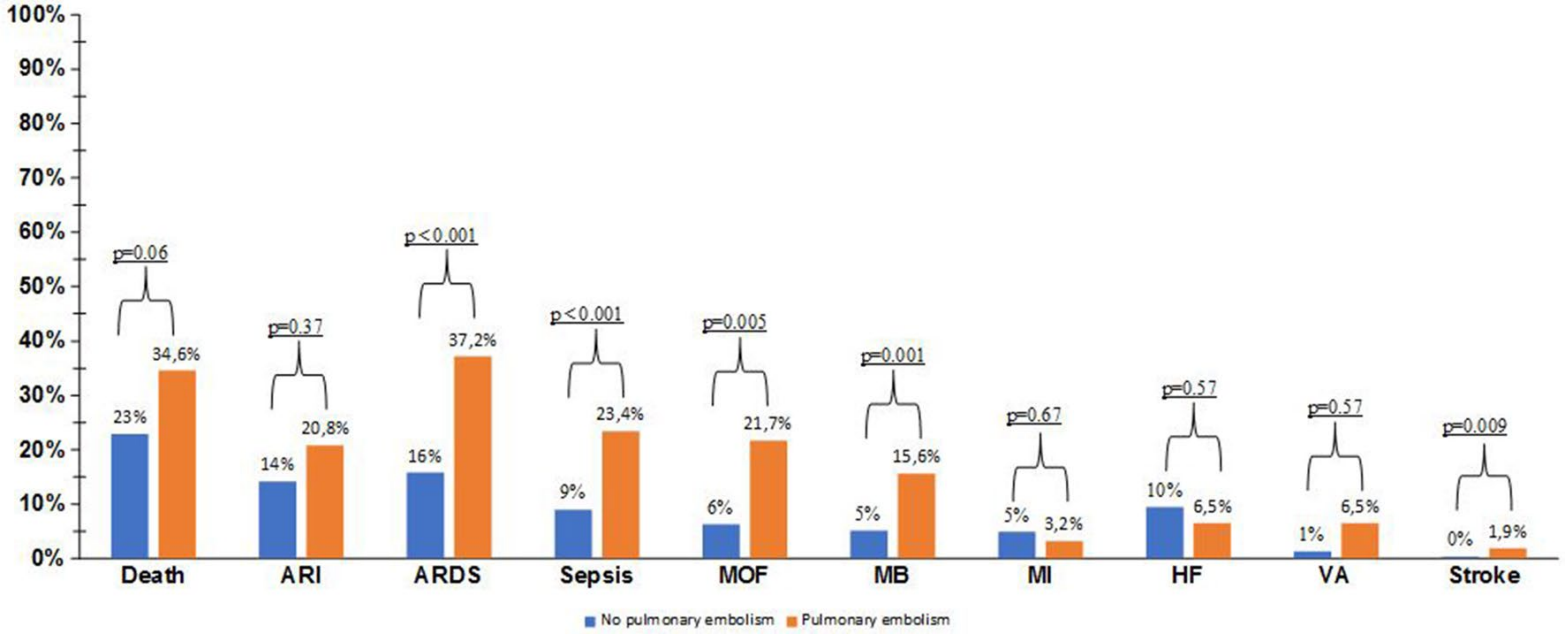
Rates of all-cause in-hospital mortality and complications, stratified by occurrence of pulmonary embolism. *ARI* acute renal insufficiency, *ARDS* acute respiratory distress syndrome, *MOF* multiorgan failure, *MB* major bleeding (requiring transfusion), *MI* myocardial infarction, *VA* ventricular arrhythmia

Compared with patients without PE, those with PE were more commonly treated with darunavir/ritonavir and tocilizumab (Table 3). The PE group was also characterized by a more frequent need of non-invasive and invasive ventilation (Table 3). Consistently, the rate of acute respiratory distress syndrome (ARDS) was more frequent in patients experiencing PE (Fig. 2).

**Table 3:** Treatment received for COVID-19 in the study population, stratified by occurrence of pulmonary embolism

	All (*n* = 689)	No PE (*n* = 637)	PE (*n* = 52)	*p* value
Medical therapy				
Lopivanir/ritonavir	184 (26.9)	169 (26.7)	15 (29.4)	0.67
Darunavir/ritonavir	168 (24.6)	148 (23.4)	20 (39.2)	0.01
Remdesivir	5 (0.7)	4 (0.6)	1 (2.0)	0.28
Corticosteroid	341 (49.9)	310 (49.0)	31 (60.8)	0.10
Tocilizumab	79 (11.5)	64 (10.1)	15 (29.4)	< 0.001
Hydroxychloroquine	574 (83.9)	527 (83.3)	47 (92.2)	0.10
Ventilation support				
Oxygen with FiO_2_ ≥ 50%	375 (55.7)	338 (54.3)	37 (74)	0.007
Non-invasive ventilation	298 (43.6)	264 (41.8)	34 (65.4)	< 0.001
Intubation	108 (15.8)	88 (13.9)	20 (38.5)	< 0.001

Data are shown as count (%)

Note that oxygen was given with a FiO_2_ ≥ 50% to both non-ventilated and ventilated patients

*sc* subcutaneous, *iv* intravenous, *FiO*_*2*_ fraction of inspired oxygen

Sepsis, multiorgan failure, acute kidney insufficiency and major bleeding (requiring transfusion) occurred more commonly in subjects with than without PE (Fig. 2). Other CV events were overall less frequent and with no significant rate differences between the two groups with the exception of stroke, which however occurred in a few subjects (Fig. 2).

During the study follow-up, 164 patients died (23.8% of total). A trend towards higher mortality was observed for patients who developed PE as compared with those who did not (34.6% vs. 22.9%, *p* = 0.06) (Fig. 2).

There were differences between patients in Lombardy and those in other regions of Italy in gender distribution, prevalence of coronary artery disease and chronic obstructive pulmonary disease, laboratory parameters on admission and treatments for COVID-19 (Supplemental Tables 3–5). Nonetheless, the rates of PE (8% vs. 6.1%, *p* = 0.41) and death (23.7% vs. 24.2%, *p* = 0.88) were similar.

### Anticoagulant therapy in patients with pulmonary embolism

Data about the timing and type of anticoagulant therapy were available for 48 of 52 (92.3%) subjects with PE. Of them, 16 (33.3%) were not on anticoagulant when PE was diagnosed. Conversely, 30 (62.5%) were treated with low-molecular-weight heparin (LMWH; 15 on a prophylactic dose, 14 on an intermediate or therapeutic dose, and 1 with dosage unknown), 1 (2.1%) with unfractionated heparin (at therapeutic dose) and 1 (2.1%) with a direct oral anticoagulant. It was not possible to understand whether anticoagulation before PE was part of the treatment strategy for COVID-19 or was due to other reasons. The patient on direct oral anticoagulant had a history of VTE. Seven subjects had prior or current cancer, and five were given thromboprophylaxis.

### Correlates of pulmonary embolism

In univariate Cox regression analysis, BMI, troponin elevation and d-dimer concentration on admission were positively associated with PE, and oxygen saturation was negatively correlated (Table 4). A significant, although numerically trivial, positive association was also found for platelet count and ferritin. In the multivariable-adjusted model, only d-dimer remained significantly associated with PE (HR 1.72, 95% CI 1.13–2.62; *p* = 0.01) (Table 4).

**Table 4:** Correlates of pulmonary embolism in the study population

	Univariate		Multivariate	
	HR (95% CI)	*p* value	HR (95% CI)	*p* value
Age	0.99 (0.97–1.01)	0.19	–	–
BMI	1.06 (1.02–1.10)	0.006	–	–
Heart failure	0.32 (0.08–1.33)	0.12	–	–
Atrial fibrillation	0.43 (0.13–1.39)	0.16	–	–
Chronic kidney disease	0.45 (0.16–1.25)	0.12	–	–
Prior anticoagulant	1.83 (0.94–3.57)	0.08	–	–
Oxygen saturation	0.95 (0.93–0.98)	0.001	–	–
White blood cell count	1.00 (0.99–1.00)	0.06	–	–
Platelet count	1.00 (1.00–1.01)	0.03	–	–
Ferritin	1.00 (1.00–1.01)	< 0.001	–	–
Aspartate transaminase	1.00 (0.99–1.00)	0.16	–	–
Lactate dehydrogenase	1.00 (0.99–1.00)	0.24	–	–
Elevated troponin	2.20 (1.22–3.95)	0.008	–	–
d-dimer on admission	2.04 (1.57–2.66)	< 0.001	1.72 (1.13–2.62)	0.01

*BMI* body mass index

The risk of PE was also increased when peak d-dimer concentration was included in the model in place of baseline d-dimer level (univariate HR 1.78, 95% CI 1.43–2.2, *p* < 0.001; multivariate HR 1.57, 95% CI 1.01–2.44; *p* = 0.05) (Supplemental Table 6).

Performing backward selection (not shown) or excluding the patients still hospitalized at the end of the follow-up (Supplemental Table 7) did not change the results of the regression analysis.

**Fig. 3:**
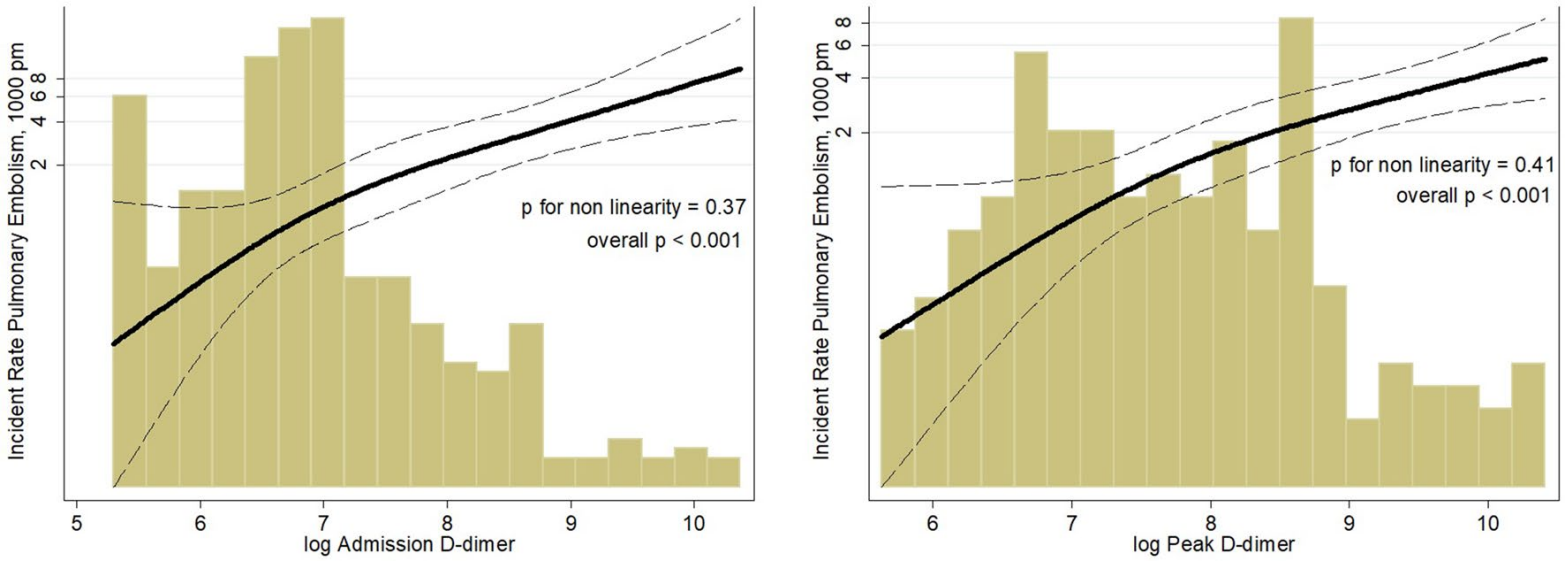
Correlation between admission (left panel) and peak (right panel) d-dimer concentrations and incidence rate of pulmonary embolism. The incidence rate is calculated per 1000 patients-month (pm). The log-transformed value corresponding to the 500 ng/mL cut-off is 6.21

The association between both baseline and peak d-dimer and the incidence of PE was linear, without an inflection point indicating a cut-off for higher risk (Fig. 3). Nevertheless, among the patients who developed PE, the concentration of d-dimer was < 500 ng/mL only in 2 (3.8%) on admission and in none at peak. Of the subjects with PE, 48 (92.3%) were ≥ 50 year-old; among them, 46 (95.8%) and 47 (97.9%) had d-dimer values on admission and at peak, respectively, above the age-adjusted threshold.

## Discussion

The main findings of this study are that PE is part of the spectrum of clinical manifestations of hospitalized COVID-19 and that d-dimer concentration is the strongest correlate of this event.

Other authors have already reported a remarkable incidence of PE in patients hospitalized for COVID-19 [15]. Most articles published so far have described subjects admitted to ICU, in whom the rate of PE was about 20% [7, 9, 10]. In 184 consecutive individuals managed in three ICU in the Netherlands, the frequency of PE was 35% [8]. In general, PE diagnosis in these series was more common than in historical cohorts of non-COVID-19 bacterial or viral acute respiratory distress syndrome, indicating that SARS-CoV-2 infection confers a specific risk of PE [7, 9]. Nonetheless, ICU patients have additional factors predisposing to VTE, such as prolonged immobilization, central venous catheters and superimposed sepsis, which may be absent in the general COVID-19 population. Indeed, according to the current evidence the frequency of PE in COVID-19 patients hospitalized in the general wards is much lower than in ICU, approximately 2–3% [11, 12].

The patients we examined were heterogeneous with respect to the severity of COVID-19 and received variable therapies. Around half had an increased respiratory rate on admission, was given antiviral drugs or corticosteroids and needed non-invasive ventilation, while 15% were intubated. However, they were managed by cardiologists and, thus, they likely represent a population with a more complex clinical profile than the one generally hospitalized for COVID-19. It is reasonable that we found a rate of PE of 7%, intermediate between those described in ICU and in general wards patients, because of these characteristics. Indeed, in a very recent analysis of a French COVID-19 cohort in which the use of invasive ventilation was around 9%, the frequency of PE was 8.3% [13].

Thus, the present work integrates the existing literature on PE in COVID-19, by providing an estimate of the rate of PE in hospitalized COVID-19 at large. It is notable, however, that features of worse COVID-19, including the rates of non-CV complications, were more common among subjects with PE.

One in five patients included in this study had PE on admission, in good agreement with the reported prevalence of PE on CTPA performed for COVID-19 at the emergency department [16]. The remaining cases of PE occurred throughout the hospitalization for COVID-19, again consistently with other investigations [7–9, 11]. Therefore, physicians should be vigilant towards the development of PE in hospitalized COVID-19 from admission to discharge. It remains to be determined whether the risk of PE is heightened also after discharge and until when.

We found that two thirds of the subjects with PE were treated with anticoagulants, mainly LMWH at various doses, before the diagnosis of PE. We could not determine whether this thromboprophylaxis was at least partially motivated by pre-existing risk factors for VTE, such as cancer. Indeed, oral anticoagulant therapy prior to admission tended to be more frequent in patients who then suffered from PE, indicating the presence of a pro-thrombotic condition. While acknowledging that this is a speculation and that the dataset analyzed here is not proper to verify it, the question arises of whether individual predisposition cooperates with SARS-CoV-2-induced inflammation and endotheliopathy in causing VTE in COVID-19. Along these lines, it is notable that thromboelastographic hypercoagulability on admission was associated with higher thrombosis rates in critically ill COVID-19 patients, despite standard drug prophylaxis [17]. It should be also taken into account that therapies for COVID-19, such as ritonavir, tocilizumab, and hydroxychloroquine, may modulate the risk of VTE, both directly, via their effects on platelets, and indirectly, through their effects on the inflammatory system and pharmacokinetic interactions with antithrombotic medications [18].

In the absence of a clear contraindication, anticoagulant thromboprophylaxis with LMWH or, secondarily, fondaparinux is now recommended during hospitalization for COVID-19 [19, 20]. However, PE appears to complicate COVID-19 in spite of such an approach, and full-dose anticoagulation has been related to improved survival at least in some patient subsets [21]. On the other side, intermediate- or therapeutic-dose anticoagulation exposes to a higher risk of bleeding, which may be also compounded by COVID-19 coagulopathy. In this regard, it is noteworthy that, in our analysis, major bleeding episodes were also three times more frequent in subjects with PE. Interestingly, hemorrhagic complications have been reported to be more common in COVID-19 than non-COVID-19 ARDS [9]. While awaiting for a better understanding of the coagulation derangement and the impact of non-conventional strategies for VTE prevention, administration of more than standard doses of anticoagulant for thromboprophylaxis should be probably reserved to selected cases of COVID-19 [20]. The impact of PE on COVID-19 prognosis is still unclear, with some authors reporting an association with mortality and others not [22]. We observed a trend for increased in-hospital mortality in patients with PE. It can be postulate that the consequences of PE in COVID-19 vary depending on the location of the clots in the pulmonary circulation and on the extent of concomitant pneumonia. Unfortunately, this type of information was not available in the dataset we evaluated, nor was it given in most other studies.

An increase in d-dimer concentrations is the most common laboratory abnormality in COVID-19 coagulopathy and reflects the activation of the coagulation cascade [23, 24]. In this analysis, d-dimer levels were fivefold higher on admission in patients with than without PE and remained substantially higher in the former than in the latter ones until discharge, when they decreased to values similar to those of the no-PE group. The same trend has been described by other authors [25] and has been attributed to ongoing thrombosis and inflammation, which eventually fade in survivors. Therefore, we argue that d-dimer was associated with PE because it is a sensitive marker of the thrombotic activation, linked to inflammation (so-called thromboinflammation), which characterizes COVID-19. Other investigators have demonstrated that d-dimer tracks with COVID-19 severity and predicts mortality [4].

In the majority of the patients evaluated here, baseline and peak d-dimer concentrations were above both the general and the age-adjusted thresholds advised by the guidelines on PE [26]. However, these cut-offs allow excluding PE when it is clinically unlikely or with low to intermediate probability, while PE in our cohort was always suspected clinically and then documented by CTPA. The meaning of d-dimer measurement in the diagnosis of PE in COVID-19 still needs to be clarified, as well as the role of other imaging techniques. Detection of RV dilation and dysfunction by transthoracic echocardiography [27] and of lower limb vein thrombosis by Doppler ultrasonography [28] may be helpful. Moreover, echography can be performed at the bedside, avoiding patient mobilization and minimizing the exposure of the healthcare personnel to SARS-CoV-2.

The major limitations of this work are those intrinsic of a retrospective analysis. Nonetheless, prospective studies were barely feasible during the SARS-CoV-2 outbreak and retrospective data are anyway informative, being COVID-19 a completely new disease. The lack of details about PE predisposing factors and site, as well as about the treatments of comorbidities, is another shortcoming, as already mentioned. The frequency of PE is probably underestimated by this study as only clinically relevant episodes leading to further diagnostic assessment were collected and the attention towards PE in COVID-19 was low in the initial phase of the outbreak in Italy. The rate of PE could have been higher if it had been systematically searched. Lastly, we could not investigate the incidence and correlates of deep venous thrombosis, which also seems to be common in COVID-19 [26].

Finally, the results presented here concern hospitalized COVID-19 patients and may be not valid for ambulatory ones, even though PE may be relevant in them too. In fact, PE may have accounted for a large proportion of unexpected out-of-hospital deaths during the COVID-19 pandemic [29].

In conclusion, PE occurred at a rate of 7% in a heterogeneous cohort of COVID-19 patients hospitalized in Cardiology Units in Italy, and d-dimer was the only correlate of this event. Efforts are needed to further characterize PE in COVID-19, with the ultimate goal of improving its prevention and management.

## Electronic supplementary material

Below is the link to the electronic supplementary material.

Supplementary material 1 (DOCX 156 kb)

## References

[1] Chen N, Zhou M, Dong X (2020). Epidemiological and clinical characteristics of 99 cases of 2019 novel coronavirus pneumonia in Wuhan, China: a descriptive study. Lancet.

[2] Richardson S, Hirsch JS, Narasimhan M (2020). Presenting characteristics, comorbidities, and outcomes among 5700 patients hospitalized with COVID-19 in the New York City area. JAMA.

[3] Guzik TJ, Mohiddin SA, Dimarco A (2020). COVID-19 and the cardiovascular system: implications for risk assessment, diagnosis, and treatment options. Cardiovasc Res.

[4] Iba T, Levy JH, Levi M, Connors JM, Thachil J (2020). Coagulopathy of Coronavirus Disease 2019. Crit Care Med.

[5] Ackermann M, Verleden SE, Kuehnel M (2020). Pulmonary vascular endothelialitis, thrombosis, and angiogenesis in Covid-19. N Engl J Med.

[6] Edler C, Schröder AS, Aepfelbacher M (2020). Dying with SARS-CoV-2 infection—an autopsy study of the first consecutive 80 cases in Hamburg, Germany. Int J Legal Med.

[7] Poissy J, Goutay J, Caplan M (2020). Pulmonary embolism in COVID-19 patients: awareness of an increased prevalence. Circulation.

[8] Klok FA, Kruip MJHA, van der Meer NJM (2020). Confirmation of the high cumulative incidence of thrombotic complications in critically ill ICU patients with COVID-19: an updated analysis. Thromb Res.

[9] Helms J, Tacquard C, Severac F (2020). High risk of thrombosis in patients with severe SARS-CoV-2 infection: a multicenter prospective cohort study. Intensive Care Med.

[10] Cui S, Chen S, Li X, Liu S, Wang F (2020). Prevalence of venous thromboembolism in patients with severe novel coronavirus pneumonia. J Thromb Haemost.

[11] Galeano-Valle F, Oblitas CM, Ferreiro-Mazón MM (2020). Antiphospholipid antibodies are not elevated in patients with severe COVID-19 pneumonia and venous thromboembolism. Thromb Res.

[12] Lodigiani C, Iapichino G, Carenzo L (2020). Venous and arterial thromboembolic complications in COVID-19 patients admitted to an academic hospital in Milan. Italy Thromb Res.

[13] Fauvel C, Weizman O, Trimaille A (2020). Pulmonary embolism in COVID-19 patients: a French multicentre cohort study. Eur Heart J.

[14] Righini M, Van Es J, Exter PLD (2014). Age-adjusted d-dimer cutoff levels to rule out pulmonary embolism: the ADJUST-PE Study. JAMA.

[15] Al-Ani F, Chehade S, Lazo-Langner A (2020). Thrombosis risk associated with COVID-19 infection. A scoping review. Thromb Res.

[16] Gervaise A, Bouzad C, Peroux E, Helissey C (2020). Acute pulmonary embolism in non-hospitalized COVID-19 patients referred to CTPA by emergency department. Eur Radiol.

[17] Mortus JR, Manek SE, Brubaker LS (2020). Thromboelastographic results and hypercoagulability syndrome in patients with Coronavirus Disease 2019 who are critically ill. JAMA Netw Open.

[18] Bikdeli B, Madhavan MV, Jimenez D (2020). COVID-19 and thrombotic or thromboembolic disease: implications for prevention, antithrombotic therapy, and follow-up: JACC state-of-the-art review. J Am Coll Cardiol.

[19] Thachil J, Tang N, Gando S (2020). ISTH interim guidance on recognition and management of coagulopathy in COVID-19. J Thromb Haemost.

[20] Moores LK, Tritschler T, Brosnahan S et al (2020) Prevention, diagnosis and treatment of venous thromboembolism in patients with COVID-19: CHEST Guideline and Expert Panel Report. Chest. 10.1016/j.chest.2020.05.559**(e-pub ahead of print)**

[21] Paranjpe I, Fuster V, Lala A et al (2020) Association of treatment dose anticoagulation with in-hospital survival among hospitalized patients with COVID-19. J Am Coll Cardiol. 10.1016/j.jacc.2020.05.001**(e-pub ahead of print)** 10.1016/j.jacc.2020.05.001 PMC7202841 32387623

[22] Connors JM, Levy JH (2020). COVID-19 and its implications for thrombosis and anticoagulation. Blood.

[23] Tang N, Li D, Wang X, Sun Z (2020). Abnormal coagulation parameters are associated with poor prognosis in patients with Novel Coronavirus pneumonia. J Thromb Haemost.

[24] Bao J, Li C, Zhang K, Kang H, Chen W, Gu B (2020) Comparative analysis of laboratory indexes of severe and non-severe patients infected with COVID-19. Clin Chim Acta. 10.1016/j.cca.2020.06.009**(e-pub ahead of print)** 10.1016/j.cca.2020.06.009 PMC7274996 32511971

[25] Yang AI, Li HM, Tao WQ et al (2020) Infection with SARS-CoV-2 causes abnormal laboratory results of multiple organs in patients. Aging (Albany NY). 10.18632/aging.103255**(e-pub ahead of print)** 10.18632/aging.103255 PMC7346014 32484453

[26] Konstantinides SV, Meyer G, Becattini C (2020). 2019 ESC Guidelines for the diagnosis and management of acute pulmonary embolism developed in collaboration with the European Respiratory Society (ERS). Eur Heart J.

[27] Szekely Y, Lichter Y, Taieb P et al (2020) The Spectrum of cardiac manifestations in Coronavirus Disease 2019 (COVID-19)—a systematic echocardiographic study. Circulation. 10.1161/CIRCULATIONAHA.120.047971**(e-pub ahead of print)** 10.1161/CIRCULATIONAHA.120.047971 PMC7382541 32469253

[28] Voicu S, Bonnin P, Stépanian A et al (2020) High prevalence of deep vein thrombosis in mechanically ventilated COVID-19 patients. J Am Coll Cardiol. 10.1016/j.jacc.2020.05.053**(e-pub ahead of print)** 10.1016/j.jacc.2020.05.053 PMC7260513 32479784

[29] Benzakoun J, Hmeydia G, Delabarde T et al (2020) Excess out-of-hospital deaths during COVID-19 outbreak: evidence of pulmonary embolism as a main determinant. Eur J Heart Fail. 10.1002/ejhf.1916**(e-pub ahead of print)** 10.1002/ejhf.1916 PMC7283748 32463538

