# Supplementary material for: Pulmonary embolism in patients with COVID-19: characteristics and outcomes in the Cardio-COVID Italy multicenter study
Source: Clin Res Cardiol. 2020 Nov 3;110(7):1020–8. doi: 10.1007/s00392-020-01766-y (PMC7607374; doi:10.1007/s00392-020-01766-y)
Supplement: Supplementary file 1 — Supplementary material 1 (DOCX 156 kb) [file 392_2020_1766_MOESM1_ESM.docx]

**Pulmonary embolism in patients with COVID-19: characteristics and outcomes in the Cardio-COVID Italy multicenter study**

Ameri P, Inciardi R, et al.

**Supplemental information**

**Supplemental Figure 1.**

**Map of Italy showing the location of the centres participating in the study.**


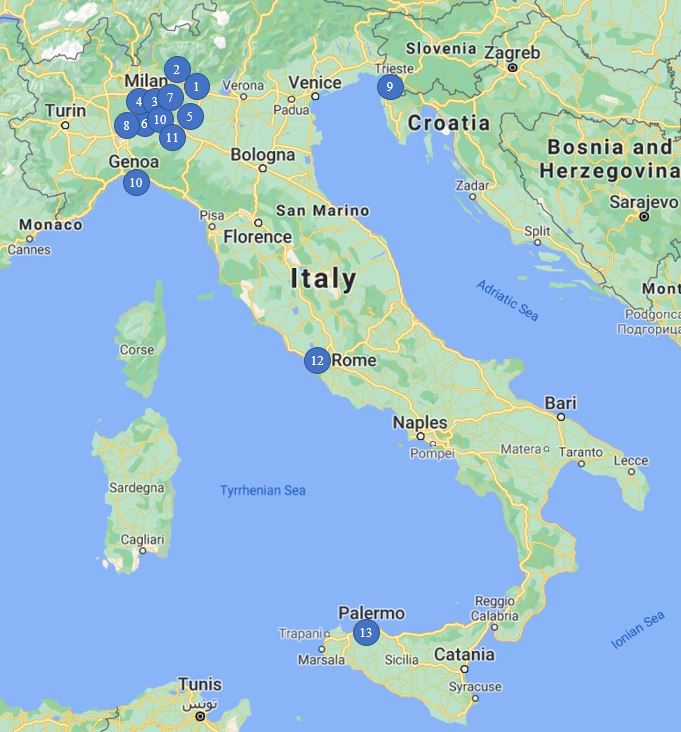


| **Study centers** | **Principal investigator** |
| --- | --- |
| 1. Institute of Cardiology, ASST Spedali Civili, Department of Medical and Surgical Specialties, Radiological Sciences, and Public Health, University of Brescia, Brescia, Italy | Prof. Marco Metra |
| 2. Division of Cardiology, Cardiovascular Department, ASST Papa Giovanni XXIII Hospital, Bergamo, Italy | Dr. Michele Senni |
| 3. Centro Cardiologico Monzino, IRCCS, Milan, Italy; Department of Clinical Sciences and Community Health, Cardiovascular Section, University of Milan, Milan, Italy. | Prof. Pier Giuseppe Agostoni |
| 4. Division of Cardiology, ASST Santi Paolo e Carlo, University of Milan, Milan, Italy. | Prof. Stefano Carugo |
| 5. Division of Cardiology, ASST Cremona, Cremona | Dr. Gian Battista Danzi |
| 6. Coronary Care Unit and Laboratory of Clinical and Experimental Cardiology- Fondazione IRCCS Policlinico San Matteo, University of Pavia, Pavia, Italy. | Dr. Sergio Leonardi |
| 7. Department of Cardiology, Policlinico di Monza, Monza, Italy. | Dr. Andrea Mortara |
| 8. Department of Cardiology, Istituti Clinici Scientifici Maugeri IRCCS, Institute of Montescano, Pavia, Italy | Dr. Maria Teresa La Rovere |
| 9. Cardiovascular Department, Azienda Sanitaria Universitaria Integrata di Trieste (ASUITS), University of Trieste, Trieste, Italy | Prof. Gianfranco Sinagra |
| 10. Cardiovascular Disease Unit, IRCCS Ospedale Policlinico San Martino, Genova, Italy | Prof. Pietro Ameri |
| 11. Heart Failure Unit, Cardiology, G. da Saliceto Hospital, Piacenza, Italy | Dr. Massimo Piepoli |
| 12. Cardiology Rehabilitation Unit, S. Raffaele IRCCS, Rome, Italy | Dr. Maurizio Volterrani |
| 13. Division of Cardiology, Buccheri La Ferla-Fatebenefratelli Hospital, Palermo, Italy | Dr. Maurizio Volterrani, Dr. Saullo |

**Supplemental Figure 2.**

**Distribution of pulmonary embolism events, as percentage of total events, throughout the hospitalization for COVID-19.**

**
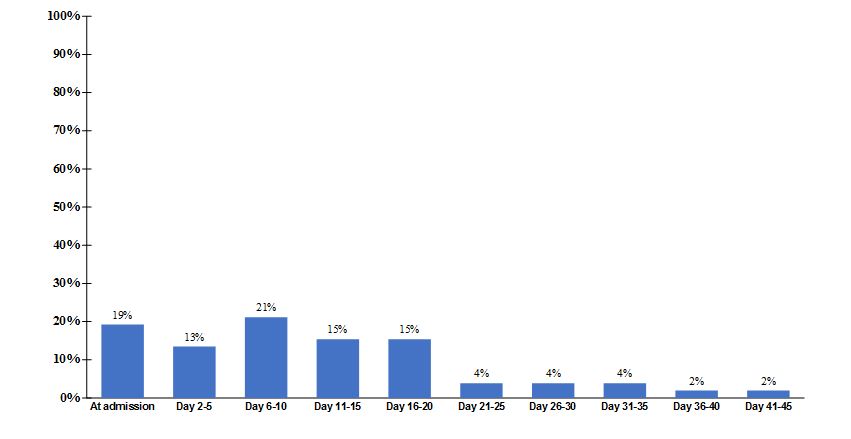
**

**Supplemental Table 1. Additional laboratory findings on admission in the study population, stratified by occurrence pulmonary embolism**.

|  | **All**  **(N. 689)** | **No PE**  **(N. 637)** | **PE**  **(N. 52)** | **P value** |
| --- | --- | --- | --- | --- |
| **Red blood cell count (x10^6/μL)** | 4.5  (4.0-4.9) | 4.5  (4.0-4.9) | 4.5  (4.1-5.1) | 0.63 |
| **Haematocrit (%)** | 39.0  (35.4-42.8) | 39.0  (35.4-42.6) | 38.6  (35.8-44.0) | 0.53 |
| **eGFR (CKD-EPI, mL/min)** | 74.2  (50.3-90.4) | 74.6  (49.8-90.6) | 71.3  (54.3-88.7) | 0.99 |
| **Serum sodium (mEq/L)** | 137  (135-140) | 137  (134-140) | 138.5  (136-141) | 0.18 |
| **Serum potassium (mEq/L)** | 3.9  (3.6-4.3) | 3.9  (3.6-4.3) | 4.1  (3.6-4.4) | 0.83 |
| **Procalcitonin (ng/mL)** | 0.2  (0.1-0.5) | 0.1  (0.1-0.4) | 0.4  (0.1-3.5) | 0.018 |
| **Bilirubin (mg/dL)** | 0.6  (0.4-0.8) | 0.6  (0.4-0.8) | 0.6  (0.4-0.8) | 0.40 |
| **Creatine phosphokinase (U/L)** | 112  (56.5-302) | 114  (54.5-302) | 92  (64-258) | 0.89 |
| **Serum albumin (g/L)** | 32  (28-36) | 32  (8-35) | 33  (26-36) | 0.89 |
| **Arterial pH** | 7.5  (7.4-7.5) | 7.5  (7.4-7.5) | 7.5  (7.4-7.5) | 0.92 |

Data shown as median (interquartile range).

eGFR: estimated glomerular filtration rate.

**Supplemental Table 2. Right ventricle-focused echocardiographic parameters on admission in the study population, stratified by pulmonary embolism.**

|  | **N. of patients with available data** | **All**  **(N. 689)** | **No PE**  **(N. 637)** | **PE (N. 52)** | **P value** |
| --- | --- | --- | --- | --- | --- |
| **RV dilation** | 242 (35.1) | 37 (15.3) | 24 (11.4) | 13 (41.9) | <0.001 |
| **RV dysfunction** | 256 (37.1) | 39 (15.2) | 29 (12.9) | 10 (31.3) | 0.007 |
| **TAPSE (mm)** | 166 (24.1) | 20.6 ± 4.4 | 20.9 ± 4.0 | 19.4 ± 5.9 | 0.12 |
| **FAC (%)** | 69 (10) | 42.2 ± 7.1 | 42.9 ± 6.5 | 39.2 ± 9.0 | 0.09 |
| **S’ TDI (cm/s)** | 80 (11.6) | 12.2 ± 2.7 | 12.5  (12 – 13.1) | 10.9  (9.1-12.8) | 0.014 |
| **sPAP (mmHg)** | 199 (28.8) | 18.3 ± 9.5 | 18.1 ± 9.1 | 19.7 ± 11.9 | 0.42 |

Data are shown as count (%), mean ± SD or median (interquartile range).

RV: right ventricle; TAPSE: tricuspid annular plane systolic excursion; FAC: fractional area change; S’ TDI: S’ tissue Doppler imaging-derived tricuspid lateral annular systolic velocity; sPAP: systolic pulmonary artery pressure.

RV dysfunction: TAPSE <17 mm or S` TDI <9.5 cm/s or enlarged RV (basal diameter >43 mm or dilation on qualitative assessment).

**Supplemental Table 3. Baseline characteristics of the study population, stratified by region of enrolment**.

|  | **All**  **(N. 689)** | **Lombardy**  **(N. 524)** | **Other regions (N. 165)** | **P value** |
| --- | --- | --- | --- | --- |
| **Age (years)** | 67.3 ± 13.2 | 67.4 ± 13.3 | 67.2 ± 13.0 | 0.91 |
| **Male gender** | 487 (69.4) | 375 (71.6) | 103 (62.4) | 0.03 |
| **BMI (kg/m^2^)** | 27.2 ± 5.3 | 27.2 ± 5.5 | 27.2 ± 5.0 | 0.9 |
| **Ever smoker** | 159 (27) | 127 (28.6) | 32 (22.2) | 0.13 |
| **Hypertension** | 398 (56.9) | 294 (56.3) | 95 (58.6) | 0.60 |
| **Dyslipidaemia** | 188 (27.5) | 145 (27.8) | 43 (26.5) | 0.75 |
| **Diabetes** | 157 (23) | 126 (24.1) | 31 (19.1) | 0.19 |
| **Heart failure** | 92 (13.5) | 72 (13.8) | 20 (12.3) | 0.64 |
| **Atrial fibrillation** | 105 (15.4) | 85 (16.3) | 20 (12.3) | 0.22 |
| **Coronary artery disease** | 143 (20.9) | 117 (22.4) | 26 (16.0) | 0.08 |
| **COPD** | 67 (9.8) | 42 (8.0) | 25 (15.4) | 0.006 |
| **Chronic kidney disease** | 127 (18.6) | 94 (18.0) | 33 (20.4) | 0.50 |
| **ACEi/ARB therapy** | 133 (20.6) | 100 (20.3) | 33 (21.4) | 0.77 |
| **Oral anticoagulant therapy** | 90 (14.1) | 74 (15.4) | 16 (10.2) | 0.10 |
| ***Direct oral anticoagulant*** | 47 (7.4) | 38 (7.9) | 9 (5.7) | 0.37 |
| ***Vitamin K antagonist*** | 48 (7.5) | 41 (8.5) | 7 (4.5) | 0.09 |
| **Statin therapy** | 176 (27.2) | 141 (28.7) | 35 (22.6) | 0.14 |
| **Fever (≥37.5 °C)** | 440 (64.1) | 332 (63.6) | 108 (65.9) | 0.60 |
| **Respiratory rate ≥22/minute** | 279 (52.0) | 189 (50.8) | 90 (54.5) | 0.42 |
| **SBP (mmHg)** | 129.6 ± 21.5 | 131.0 ± 21.8 | 129.2 ± 21.4 | 0.34 |
| **Heart rate (bpm)** | 86.6 ± 18.1 | 86.5 ± 18.0 | 87.2 ± 18.4 | 0.66 |
| **Oxygen saturation (%)** | 90.5 ± 7.6 | 90.4 ± 7.8 | 90.9 ± 6.6 | 0.40 |
| **LV ejection fraction (%)** | 52.5 ± 11.3 | 52.3 ± 12.2 | 53.0 ± 9.0 | 0.61 |

Data are shown as count (%), mean ± SD or median (interquartile range).

BMI: body mass index; COPD: chronic obstructive pulmonary disease; ACEi: angiotensin-converting enzyme inhibitor; ARB: angiotensin receptor blocker; SBP systolic blood pressure; LV: left ventricular.

**Supplemental Table 4. Main laboratory findings on admission in the study population, stratified by region of enrolment**.

|  | **All  (N. 689)** | **Lombardy**  **(N. 524)** | **Other regions**  **(N. 165)** | **P value** |
| --- | --- | --- | --- | --- |
| **Haemoglobin (g/dL)** | 13.4  (11.8-14.4) | 13.2  (11.7-14.3) | 13.7  (12.2-14.8) | 0.007 |
| **White blood cell count (/μL)** | 6,760  (4,990-9,320) | 6,945  (5,165-9,270) | 6,300  (4,590-9,440) | 0.09 |
| **Lymphocyte count (/μL)** | 921.5  (620-1,300) | 920  (600-1,296) | 995  (690-1,300) | 0.69 |
| **Platelet count (x10^3/μL)** | 204  (155-270) | 209  (157-273) | 195.5  (147.5-265) | 0.15 |
| **Serum creatinine (mg/dL)** | 1.0  (0.8-1.3) | 1.0  (0.8-1.4) | 0.9  (0.8-1.1) | 0.007 |
| **CRP (mg/dL)** | 233.5  (108.5-353.5) | 237.0  (111.0-349.0) | 198.0  (83.0-379.0) | 0.69 |
| **Ferritin (μg/L)** | 698.5  (374-1,473) | 722  (364-1,562) | 674  (396-1,376) | 0.44 |
| **Aspartate transaminase (U/L)** | 40  (26-64) | 42  (26-68) | 38  (24-56) | 0.06 |
| **Lactate dehydrogenase (U/L)** | 362  (252-520) | 384.5  (257.5-587.0) | 311.0  (243.0-425.0) | <0.001 |
| **D-dimer (ng/mL)** | 868  (439-1,703) | 818  (394-1,805) | 970  (515-1,500) | 0.21 |
| **INR** | 1.1  (1.0-1.2) | 1.1  (1.0-1.2) | 1.2  (1.1-1.3) | <0.001 |
| **Elevated troponin** | 278 (45.3) | 228 (44.3) | 44 (50.6) | 0.27 |
| **NT-proBNP (pg/mL)** | 341  (96-1,323) | 417  (134-2,403) | 187  (71-920) | 0.013 |

Data shown as median (interquartile range) or, for the frequency of elevated troponin, count (%).

CRP: C-reactive protein; INR: International Normalized Ratio: NT-proBNP: N-terminal pro-B-type natriuretic peptide.

**Supplemental Table 5. Treatment received for COVID-19 in the study population, stratified by region of enrolment.**

|  | **All**  **(N. 689)** | **Lombardy**  **(N. 524)** | **Other regions**  **(N. 165)** | **P value** |
| --- | --- | --- | --- | --- |
| ***Medical therapy*** |  |  |  |  |
| **Lopivanir/Ritonavir** | 184 (26.9) | 148 (28.5) | 36 (22.0) | 0.10 |
| **Darunavir/Ritonavir** | 168 (24.6) | 155 (29.8) | 13 (7.9) | <0.001 |
| **Remdesivir** | 5 (0.7) | 5 (1.0) | 0 (0.0) | 0.21 |
| **Corticosteroid** | 341 (49.9) | 212 (40.8) | 129 (78.7) | <0.001 |
| **Tocilizumab** | 79 (11.5) | 38 (7.3) | 41 (25.0) | <0.001 |
| **Hydroxychloroquine** | 574 (83.9) | 431 (82.9) | 143 (87.2) | 0.19 |
| ***Ventilation support*** |  |  |  |  |
| **Oxygen with FiO_2_** **≥50%** | 375 (55.7) | 257 (50.6) | 118 (71.5) | <0.001 |
| **Non-invasive ventilation** | 298 (43.6) | 198 (38.2) | 100 (61) | <0.001 |
| **Intubation** | 108 (15.8) | 78 (15) | 30 (18.3) | 0.31 |

Data are shown as count (%).

Note that oxygen was given with a FiO2 ≥50% to both non-ventilated and ventilated patients.

sc: subcutaneous; iv: intravenous; FiO_2_: fraction of inspired oxygen.

**Supplemental Table 6. Correlates of pulmonary embolism in the study population, after substituting peak D-dimer for D-dimer on admission.**

|  | **Univariate** | | **Multivariate** | |
| --- | --- | --- | --- | --- |
|  | HR (95%CI) | P value | HR (95%CI) | P value |
| **Age** | 0.99  (0.97-1.01) | 0.19 | - | - |
| **BMI** | 1.06  (1.02-1.10) | 0.006 | - | - |
| **Heart failure** | 0.32  (0.08-1.33) | 0.12 | - | - |
| **Atrial fibrillation** | 0.43  (0.13-1.39) | 0.16 | - | - |
| **Chronic kidney disease** | 0.45  (0.16-1.25) | 0.12 | - | - |
| **Prior anticoagulant** | 1.83  (0.94-3.57) | 0.08 | - | - |
| **Oxygen saturation** | 0.95  (0.93-0.98) | 0.001 | - | - |
| **White blood cell count** | 1.00  (0.99-1.00) | 0.06 | - | - |
| **Platelet count** | 1.00  (1.00-1.01) | 0.03 | - | - |
| **Ferritin** | 1.00  (1.00-1.01) | <0.001 | - | - |
| **Aspartate transaminase** | 1.00  (0.99-1.00) | 0.16 | - | - |
| **Lactate dehydrogenase** | 1.00  (0.99-1.00) | 0.24 | - | - |
| **Elevated troponin** | 2.20  (1.22-3.95) | 0.008 | - | - |
| **Peak D-dimer** | 1.78  (1.43-2.20) | <0.001 | 1.57  (1.01-2.45) | 0.05 |

BMI: body mass index.

**Supplemental Table 7. Correlates of pulmonary embolism in closed cases.**

|  | **Univariate** | | **Multivariate** | |
| --- | --- | --- | --- | --- |
|  | HR (95% CI) | P value | HR (95% CI) | P value |
| **Age** | 0.99  (0.96-1.01) | 0.22 | - | - |
| **BMI** | 1.08  (1.03-1.13) | 0.001 | - | - |
| **Heart failure** | 0.37  (0.09-1.53) | 0.17 | - | - |
| **Atrial fibrillation** | 0.51  (0.16-1.64) | 0.26 | - | - |
| **Chronic kidney disease** | 0.49  (0.17-1.36) | 0.17 | - | - |
| **Prior anticoagulant** | 1.69  (0.78-3.65) | 0.18 | - | - |
| **Oxygen saturation** | 0.95  (0.93-0.98) | 0.002 | - | - |
| **White blood cell count** | 1.00  (1.00-1.01) | 0.06 | - | - |
| **Platelet count** | 1.00  (1-1.01) | 0.05 | - | - |
| **Ferritin** | 1.00  (1.00-1.01) | <0.001 | - | - |
| **Aspartate transaminase** | 1.00  (0.99-1.00) | 0.15 | - | - |
| **Lactate dehydrogenase** | 1.00  (0.99-1.00) | 0.23 | - | - |
| **Elevated troponin** | 2.16  (1.11-4.18) | 0.02 | - | - |
| **D-dimer on admission** | 2.18  (1.66-2.87) | < 0.001 | 1.60  (1.07-2.40) | 0.02 |

BMI: body mass index.
